# Supplementary material for: A UMLS-based spell checker for natural language processing in vaccine safety
Source: BMC Med Inform Decis Mak. 2007 Feb 12;7:3. doi: 10.1186/1472-6947-7-3 (PMC1805499; doi:10.1186/1472-6947-7-3)
Supplement: Additional file 13 — RAP application directory. Sets up directory for RAP (RDF application for PHP) source code [file 1472-6947-7-3-S13.gz › rap/doc/phpdoc/elementindex_default.html]

xml version="1.0" encoding="iso-8859-1"?


## [default] element index

### Package indexes

- infModel
- model
- ontModel
- rdql
- resModel
- syntax
- utility

All elements
  

a
b
c
d
e
f
g
h
i
j
k
l
m
n
o
p
r
s
t
u
v
w
x
y

a

top

ABOUT
:   RDF\_RES::ABOUT() in RDF\_RES.php

ABOUT
:   RDF::ABOUT() in RDF\_C.php

ABOUT\_EACH
:   RDF\_RES::ABOUT\_EACH() in RDF\_RES.php

ABOUT\_EACH
:   RDF::ABOUT\_EACH() in RDF\_C.php

ABOUT\_EACH\_PREFIX
:   RDF\_RES::ABOUT\_EACH\_PREFIX() in RDF\_RES.php

ABOUT\_EACH\_PREFIX
:   RDF::ABOUT\_EACH\_PREFIX() in RDF\_C.php

ABSTRACT\_
:   DC\_RES::ABSTRACT\_() in DC\_RES.php

ABSTRACT\_
:   DC::ABSTRACT\_() in DC\_C.php

ACCESS\_RIGHTS
:   DC::ACCESS\_RIGHTS() in DC\_C.php

ACCESS\_RIGHTS
:   DC\_RES::ACCESS\_RIGHTS() in DC\_RES.php

ACCOUNT\_NAME
:   FOAF\_RES::ACCOUNT\_NAME() in FOAF\_RES.php

ACCOUNT\_NAME
:   FOAF::ACCOUNT\_NAME() in FOAF\_C.php

ACCOUNT\_SERVICE\_HOMEPAGE
:   FOAF\_RES::ACCOUNT\_SERVICE\_HOMEPAGE() in FOAF\_RES.php

ACCOUNT\_SERVICE\_HOMEPAGE
:   FOAF::ACCOUNT\_SERVICE\_HOMEPAGE() in FOAF\_C.php

ADODB\_DB\_DRIVER
:   ADODB\_DB\_DRIVER in constants.php

ADODB\_DB\_HOST
:   ADODB\_DB\_HOST in constants.php

ADODB\_DB\_NAME
:   ADODB\_DB\_NAME in constants.php

ADODB\_DB\_PASSWORD
:   ADODB\_DB\_PASSWORD in constants.php

ADODB\_DB\_USER
:   ADODB\_DB\_USER in constants.php

ADODB\_DEBUG\_MODE
:   ADODB\_DEBUG\_MODE in constants.php

ADR
:   VCARD::ADR() in VCARD\_C.php

ADR
:   VCARD\_RES::ADR() in VCARD\_RES.php

ADRPROPERTIES
:   VCARD::ADRPROPERTIES() in VCARD\_C.php

ADRPROPERTIES
:   VCARD\_RES::ADRPROPERTIES() in VCARD\_RES.php

ADRTYPES
:   VCARD::ADRTYPES() in VCARD\_C.php

ADRTYPES
:   VCARD\_RES::ADRTYPES() in VCARD\_RES.php

AGENT
:   VCARD\_RES::AGENT() in VCARD\_RES.php

AGENT
:   FOAF\_RES::AGENT() in FOAF\_RES.php

AGENT
:   VCARD::AGENT() in VCARD\_C.php

AGENT
:   FOAF::AGENT() in FOAF\_C.php

AIM\_CHAT\_ID
:   FOAF::AIM\_CHAT\_ID() in FOAF\_C.php

AIM\_CHAT\_ID
:   FOAF\_RES::AIM\_CHAT\_ID() in FOAF\_RES.php

ALL\_DIFFERENT
:   OWL\_RES::ALL\_DIFFERENT() in OWL\_RES.php

ALL\_DIFFERENT
:   OWL::ALL\_DIFFERENT() in OWL\_C.php

ALL\_VALUES\_FROM
:   OWL::ALL\_VALUES\_FROM() in OWL\_C.php

ALL\_VALUES\_FROM
:   OWL\_RES::ALL\_VALUES\_FROM() in OWL\_RES.php

ALT
:   RDF\_RES::ALT() in RDF\_RES.php

ALT
:   RDF::ALT() in RDF\_C.php

ALTERNATIVE
:   DC\_RES::ALTERNATIVE() in DC\_RES.php

ALTERNATIVE
:   DC::ALTERNATIVE() in DC\_C.php

ANNOTATION\_PROPERTY
:   OWL::ANNOTATION\_PROPERTY() in OWL\_C.php

ANNOTATION\_PROPERTY
:   OWL\_RES::ANNOTATION\_PROPERTY() in OWL\_RES.php

ATOM
:   ATOM in ATOM\_C.php

ATOM\_CONSTRUCT
:   ATOM\_RES::ATOM\_CONSTRUCT() in ATOM\_RES.php

ATOM\_CONSTRUCT
:   ATOM::ATOM\_CONSTRUCT() in ATOM\_C.php

ATOM\_NS
:   ATOM\_NS in constants.php

ATOM\_RES
:   ATOM\_RES in ATOM\_RES.php

AUDIENCE
:   DC\_RES::AUDIENCE() in DC\_RES.php

AUDIENCE
:   DC::AUDIENCE() in DC\_C.php

AUTHOR
:   ATOM\_RES::AUTHOR() in ATOM\_RES.php

AUTHOR
:   ATOM::AUTHOR() in ATOM\_C.php

AVAILABLE
:   DC\_RES::AVAILABLE() in DC\_RES.php

AVAILABLE
:   DC::AVAILABLE() in DC\_C.php

ATOM\_C.php
:   ATOM\_C.php in ATOM\_C.php

ATOM\_RES.php
:   ATOM\_RES.php in ATOM\_RES.php

b

top

BACKWARD\_COMPATIBLE\_WITH
:   OWL\_RES::BACKWARD\_COMPATIBLE\_WITH() in OWL\_RES.php

BACKWARD\_COMPATIBLE\_WITH
:   OWL::BACKWARD\_COMPATIBLE\_WITH() in OWL\_C.php

BAG
:   RDF\_RES::BAG() in RDF\_RES.php

BAG
:   RDF::BAG() in RDF\_C.php

BAG\_ID
:   RDF::BAG\_ID() in RDF\_C.php

BAG\_ID
:   RDF\_RES::BAG\_ID() in RDF\_RES.php

BASED\_NEAR
:   FOAF::BASED\_NEAR() in FOAF\_C.php

BASED\_NEAR
:   FOAF\_RES::BASED\_NEAR() in FOAF\_RES.php

BDAY
:   VCARD\_RES::BDAY() in VCARD\_RES.php

BDAY
:   VCARD::BDAY() in VCARD\_C.php

BIBLIOGRAPHIC\_CITATION
:   DC::BIBLIOGRAPHIC\_CITATION() in DC\_C.php

BIBLIOGRAPHIC\_CITATION
:   DC\_RES::BIBLIOGRAPHIC\_CITATION() in DC\_RES.php

BNODE\_PREFIX
:   BNODE\_PREFIX in constants.php

BOX
:   DC::BOX() in DC\_C.php

BOX
:   DC\_RES::BOX() in DC\_RES.php

c

top

CARDINALITY
:   OWL\_RES::CARDINALITY() in OWL\_RES.php

CARDINALITY
:   OWL::CARDINALITY() in OWL\_C.php

CATEGORIES
:   VCARD\_RES::CATEGORIES() in VCARD\_RES.php

CATEGORIES
:   VCARD::CATEGORIES() in VCARD\_C.php

CHANNEL
:   RSS::CHANNEL() in RSS\_C.php

CHANNEL
:   RSS\_RES::CHANNEL() in RSS\_RES.php

COLLECTION
:   DC\_RES::COLLECTION() in DC\_RES.php

COLLECTION
:   DC::COLLECTION() in DC\_C.php

COMMENT
:   RDFS\_RES::COMMENT() in RDFS\_RES.php

COMMENT
:   RDFS::COMMENT() in RDFS\_C.php

COMPLEMENT\_OF
:   OWL\_RES::COMPLEMENT\_OF() in OWL\_RES.php

COMPLEMENT\_OF
:   OWL::COMPLEMENT\_OF() in OWL\_C.php

CONFORMS\_TO
:   DC\_RES::CONFORMS\_TO() in DC\_RES.php

CONFORMS\_TO
:   DC::CONFORMS\_TO() in DC\_C.php

constants.php
:   constants.php in constants.php

CONTAINER
:   RDFS\_RES::CONTAINER() in RDFS\_RES.php

CONTAINER
:   RDFS::CONTAINER() in RDFS\_C.php

CONTAINER\_MEMBERSHIP\_PROPERTY
:   RDFS::CONTAINER\_MEMBERSHIP\_PROPERTY() in RDFS\_C.php

CONTAINER\_MEMBERSHIP\_PROPERTY
:   RDFS\_RES::CONTAINER\_MEMBERSHIP\_PROPERTY() in RDFS\_RES.php

CONTENT
:   ATOM::CONTENT() in ATOM\_C.php

CONTENT
:   ATOM\_RES::CONTENT() in ATOM\_RES.php

CONTRIBUTOR
:   ATOM::CONTRIBUTOR() in ATOM\_C.php

CONTRIBUTOR
:   ATOM\_RES::CONTRIBUTOR() in ATOM\_RES.php

CONTRIBUTOR
:   DC::CONTRIBUTOR() in DC\_C.php

CONTRIBUTOR
:   DC\_RES::CONTRIBUTOR() in DC\_RES.php

COPYRIGHT
:   ATOM::COPYRIGHT() in ATOM\_C.php

COPYRIGHT
:   ATOM\_RES::COPYRIGHT() in ATOM\_RES.php

COUNTRY
:   VCARD::COUNTRY() in VCARD\_C.php

COUNTRY
:   VCARD\_RES::COUNTRY() in VCARD\_RES.php

COVERAGE
:   DC\_RES::COVERAGE() in DC\_RES.php

COVERAGE
:   DC::COVERAGE() in DC\_C.php

CREATED
:   DC::CREATED() in DC\_C.php

CREATED
:   DC\_RES::CREATED() in DC\_RES.php

CREATED
:   ATOM\_RES::CREATED() in ATOM\_RES.php

CREATED
:   ATOM::CREATED() in ATOM\_C.php

CREATE\_MODEL\_WITHOUT\_DUPLICATES
:   CREATE\_MODEL\_WITHOUT\_DUPLICATES in constants.php

CREATOR
:   DC::CREATOR() in DC\_C.php

CREATOR
:   DC\_RES::CREATOR() in DC\_RES.php

CURRENT\_PROJECT
:   FOAF::CURRENT\_PROJECT() in FOAF\_C.php

CURRENT\_PROJECT
:   FOAF\_RES::CURRENT\_PROJECT() in FOAF\_RES.php

d

top

DATASET
:   DC\_RES::DATASET() in DC\_RES.php

DATASET
:   DC::DATASET() in DC\_C.php

DATATYPE
:   RDF::DATATYPE() in RDF\_C.php

DATATYPE
:   RDFS\_RES::DATATYPE() in RDFS\_RES.php

DATATYPE
:   RDFS::DATATYPE() in RDFS\_C.php

DATATYPE
:   OWL::DATATYPE() in OWL\_C.php

DATATYPE
:   OWL\_RES::DATATYPE() in OWL\_RES.php

DATATYPE
:   RDF\_RES::DATATYPE() in RDF\_RES.php

DATATYPE\_PROPERTY
:   OWL\_RES::DATATYPE\_PROPERTY() in OWL\_RES.php

DATATYPE\_PROPERTY
:   OWL::DATATYPE\_PROPERTY() in OWL\_C.php

DATATYPE\_RESTRICTION
:   OWL::DATATYPE\_RESTRICTION() in OWL\_C.php

DATATYPE\_RESTRICTION
:   OWL\_RES::DATATYPE\_RESTRICTION() in OWL\_RES.php

DATATYPE\_SHORTCUT\_PREFIX
:   DATATYPE\_SHORTCUT\_PREFIX in constants.php

DATA\_RANGE
:   OWL\_RES::DATA\_RANGE() in OWL\_RES.php

DATA\_RANGE
:   OWL::DATA\_RANGE() in OWL\_C.php

DATE
:   DC\_RES::DATE() in DC\_RES.php

DATE
:   DC::DATE() in DC\_C.php

DATE\_ACCEPTED
:   DC\_RES::DATE\_ACCEPTED() in DC\_RES.php

DATE\_ACCEPTED
:   DC::DATE\_ACCEPTED() in DC\_C.php

DATE\_COPYRIGHTED
:   DC\_RES::DATE\_COPYRIGHTED() in DC\_RES.php

DATE\_COPYRIGHTED
:   DC::DATE\_COPYRIGHTED() in DC\_C.php

DATE\_SUBMITTED
:   DC\_RES::DATE\_SUBMITTED() in DC\_RES.php

DATE\_SUBMITTED
:   DC::DATE\_SUBMITTED() in DC\_C.php

DBMODEL
:   DBMODEL in constants.php

DC
:   DC in DC\_C.php

DCMITYPE\_NS
:   DCMITYPE\_NS in constants.php

DCMI\_TYPE
:   DC::DCMI\_TYPE() in DC\_C.php

DCMI\_TYPE
:   DC\_RES::DCMI\_TYPE() in DC\_RES.php

DCTERM\_NS
:   DCTERM\_NS in constants.php

DC\_NS
:   DC\_NS in constants.php

DC\_RES
:   DC\_RES in DC\_RES.php

DEFAULT\_ALGORITHM
:   DEFAULT\_ALGORITHM in constants.php

DEFAULT\_ENCODING
:   DEFAULT\_ENCODING in constants.php

DEPICTION
:   FOAF\_RES::DEPICTION() in FOAF\_RES.php

DEPICTION
:   FOAF::DEPICTION() in FOAF\_C.php

DEPICTS
:   FOAF::DEPICTS() in FOAF\_C.php

DEPICTS
:   FOAF\_RES::DEPICTS() in FOAF\_RES.php

DEPRECATED\_CLASS
:   OWL::DEPRECATED\_CLASS() in OWL\_C.php

DEPRECATED\_CLASS
:   OWL\_RES::DEPRECATED\_CLASS() in OWL\_RES.php

DEPRECATED\_PROPERTY
:   OWL\_RES::DEPRECATED\_PROPERTY() in OWL\_RES.php

DEPRECATED\_PROPERTY
:   OWL::DEPRECATED\_PROPERTY() in OWL\_C.php

DESCRIPTION
:   DC::DESCRIPTION() in DC\_C.php

DESCRIPTION
:   DC\_RES::DESCRIPTION() in DC\_RES.php

DESCRIPTION
:   RDF::DESCRIPTION() in RDF\_C.php

DESCRIPTION
:   RSS::DESCRIPTION() in RSS\_C.php

DESCRIPTION
:   RSS\_RES::DESCRIPTION() in RSS\_RES.php

DESCRIPTION
:   RDF\_RES::DESCRIPTION() in RDF\_RES.php

DIFFERENT\_FROM
:   OWL\_RES::DIFFERENT\_FROM() in OWL\_RES.php

DIFFERENT\_FROM
:   OWL::DIFFERENT\_FROM() in OWL\_C.php

DISJOINT\_WITH
:   OWL\_RES::DISJOINT\_WITH() in OWL\_RES.php

DISJOINT\_WITH
:   OWL::DISJOINT\_WITH() in OWL\_C.php

DISTINCT\_MEMBERS
:   OWL\_RES::DISTINCT\_MEMBERS() in OWL\_RES.php

DISTINCT\_MEMBERS
:   OWL::DISTINCT\_MEMBERS() in OWL\_C.php

DNA\_CHECKSUM
:   FOAF\_RES::DNA\_CHECKSUM() in FOAF\_RES.php

DNA\_CHECKSUM
:   FOAF::DNA\_CHECKSUM() in FOAF\_C.php

DOCUMENT
:   FOAF::DOCUMENT() in FOAF\_C.php

DOCUMENT
:   FOAF\_RES::DOCUMENT() in FOAF\_RES.php

DOMAIN
:   RDFS\_RES::DOMAIN() in RDFS\_RES.php

DOMAIN
:   RDFS::DOMAIN() in RDFS\_C.php

DBase.php
:   DBase.php in DBase.php

DC\_C.php
:   DC\_C.php in DC\_C.php

DC.php
:   DC.php in DC.php

DC\_RES.php
:   DC\_RES.php in DC\_RES.php

e

top

EDUCATION\_LEVEL
:   DC\_RES::EDUCATION\_LEVEL() in DC\_RES.php

EDUCATION\_LEVEL
:   DC::EDUCATION\_LEVEL() in DC\_C.php

EMAIL
:   VCARD\_RES::EMAIL() in VCARD\_RES.php

EMAIL
:   ATOM\_RES::EMAIL() in ATOM\_RES.php

EMAIL
:   ATOM::EMAIL() in ATOM\_C.php

EMAIL
:   VCARD::EMAIL() in VCARD\_C.php

EMAILTYPES
:   VCARD\_RES::EMAILTYPES() in VCARD\_RES.php

EMAILTYPES
:   VCARD::EMAILTYPES() in VCARD\_C.php

ENTRY
:   ATOM\_RES::ENTRY() in ATOM\_RES.php

ENTRY
:   ATOM::ENTRY() in ATOM\_C.php

EQUIVALENT\_CLASS
:   OWL\_RES::EQUIVALENT\_CLASS() in OWL\_RES.php

EQUIVALENT\_CLASS
:   OWL::EQUIVALENT\_CLASS() in OWL\_C.php

EQUIVALENT\_PROPERTY
:   OWL\_RES::EQUIVALENT\_PROPERTY() in OWL\_RES.php

EQUIVALENT\_PROPERTY
:   OWL::EQUIVALENT\_PROPERTY() in OWL\_C.php

EVENT
:   DC\_RES::EVENT() in DC\_RES.php

EVENT
:   DC::EVENT() in DC\_C.php

EXTADD
:   VCARD::EXTADD() in VCARD\_C.php

EXTADD
:   VCARD\_RES::EXTADD() in VCARD\_RES.php

EXTENT
:   DC\_RES::EXTENT() in DC\_RES.php

EXTENT
:   DC::EXTENT() in DC\_C.php

f

top

FAMILY
:   VCARD\_RES::FAMILY() in VCARD\_RES.php

FAMILY
:   VCARD::FAMILY() in VCARD\_C.php

FAMILY\_NAME
:   FOAF\_RES::FAMILY\_NAME() in FOAF\_RES.php

FAMILY\_NAME
:   FOAF::FAMILY\_NAME() in FOAF\_C.php

FEED
:   ATOM::FEED() in ATOM\_C.php

FEED
:   ATOM\_RES::FEED() in ATOM\_RES.php

FIRST
:   RDF\_RES::FIRST() in RDF\_RES.php

FIRST
:   RDF::FIRST() in RDF\_C.php

FIRST\_NAME
:   FOAF::FIRST\_NAME() in FOAF\_C.php

FIRST\_NAME
:   FOAF\_RES::FIRST\_NAME() in FOAF\_RES.php

FIX\_BLANKNODES
:   FIX\_BLANKNODES in constants.php

FN
:   VCARD\_RES::FN() in VCARD\_RES.php

FN
:   VCARD::FN() in VCARD\_C.php

FOAF
:   FOAF in FOAF\_C.php

FOAF\_NS
:   FOAF\_NS in constants.php

FOAF\_RES
:   FOAF\_RES in FOAF\_RES.php

FORMAT
:   DC::FORMAT() in DC\_C.php

FORMAT
:   DC\_RES::FORMAT() in DC\_RES.php

FUNCTIONAL\_PROPERTY
:   OWL::FUNCTIONAL\_PROPERTY() in OWL\_C.php

FUNCTIONAL\_PROPERTY
:   OWL\_RES::FUNCTIONAL\_PROPERTY() in OWL\_RES.php

FUNDED\_BY
:   FOAF::FUNDED\_BY() in FOAF\_C.php

FUNDED\_BY
:   FOAF\_RES::FUNDED\_BY() in FOAF\_RES.php

FOAF\_C.php
:   FOAF\_C.php in FOAF\_C.php

FOAF.php
:   FOAF.php in FOAF.php

FOAF\_RES.php
:   FOAF\_RES.php in FOAF\_RES.php

g

top

GEEKCODE
:   FOAF::GEEKCODE() in FOAF\_C.php

GEEKCODE
:   FOAF\_RES::GEEKCODE() in FOAF\_RES.php

GENDER
:   FOAF::GENDER() in FOAF\_C.php

GENDER
:   FOAF\_RES::GENDER() in FOAF\_RES.php

GENERAL\_PREFIX\_BASE
:   GENERAL\_PREFIX\_BASE in constants.php

GENERATOR
:   ATOM\_RES::GENERATOR() in ATOM\_RES.php

GENERATOR
:   ATOM::GENERATOR() in ATOM\_C.php

GEO
:   VCARD::GEO() in VCARD\_C.php

GEO
:   VCARD\_RES::GEO() in VCARD\_RES.php

GIVEN
:   VCARD::GIVEN() in VCARD\_C.php

GIVEN
:   VCARD\_RES::GIVEN() in VCARD\_RES.php

GIVENNAME
:   FOAF::GIVENNAME() in FOAF\_C.php

GIVENNAME
:   FOAF\_RES::GIVENNAME() in FOAF\_RES.php

GROUP
:   VCARD\_RES::GROUP() in VCARD\_RES.php

GROUP
:   FOAF\_RES::GROUP() in FOAF\_RES.php

GROUP
:   FOAF::GROUP() in FOAF\_C.php

GROUP
:   VCARD::GROUP() in VCARD\_C.php

h

top

HAS\_CHILD
:   ATOM\_RES::HAS\_CHILD() in ATOM\_RES.php

HAS\_CHILD
:   ATOM::HAS\_CHILD() in ATOM\_C.php

HAS\_ENTRY
:   ATOM\_RES::HAS\_ENTRY() in ATOM\_RES.php

HAS\_ENTRY
:   ATOM::HAS\_ENTRY() in ATOM\_C.php

HAS\_FORMAT
:   DC\_RES::HAS\_FORMAT() in DC\_RES.php

HAS\_FORMAT
:   DC::HAS\_FORMAT() in DC\_C.php

HAS\_LINK
:   ATOM\_RES::HAS\_LINK() in ATOM\_RES.php

HAS\_LINK
:   ATOM::HAS\_LINK() in ATOM\_C.php

HAS\_PART
:   DC\_RES::HAS\_PART() in DC\_RES.php

HAS\_PART
:   DC::HAS\_PART() in DC\_C.php

HAS\_TITLE
:   ATOM\_RES::HAS\_TITLE() in ATOM\_RES.php

HAS\_TITLE
:   ATOM::HAS\_TITLE() in ATOM\_C.php

HAS\_VALUE
:   OWL::HAS\_VALUE() in OWL\_C.php

HAS\_VALUE
:   OWL\_RES::HAS\_VALUE() in OWL\_RES.php

HAS\_VERSION
:   DC\_RES::HAS\_VERSION() in DC\_RES.php

HAS\_VERSION
:   DC::HAS\_VERSION() in DC\_C.php

HIDE\_ADVERTISE
:   HIDE\_ADVERTISE in constants.php

HOLDS\_ACCOUNT
:   FOAF::HOLDS\_ACCOUNT() in FOAF\_C.php

HOLDS\_ACCOUNT
:   FOAF\_RES::HOLDS\_ACCOUNT() in FOAF\_RES.php

HOMEPAGE
:   FOAF::HOMEPAGE() in FOAF\_C.php

HOMEPAGE
:   FOAF\_RES::HOMEPAGE() in FOAF\_RES.php

HREF
:   ATOM::HREF() in ATOM\_C.php

HREF
:   ATOM\_RES::HREF() in ATOM\_RES.php

HTML\_TABLE\_BNODE\_COLOR
:   HTML\_TABLE\_BNODE\_COLOR in constants.php

HTML\_TABLE\_HEADER\_COLOR
:   HTML\_TABLE\_HEADER\_COLOR in constants.php

HTML\_TABLE\_LITERAL\_COLOR
:   HTML\_TABLE\_LITERAL\_COLOR in constants.php

HTML\_TABLE\_NS\_ROW\_COLOR0
:   HTML\_TABLE\_NS\_ROW\_COLOR0 in constants.php

HTML\_TABLE\_NS\_ROW\_COLOR1
:   HTML\_TABLE\_NS\_ROW\_COLOR1 in constants.php

HTML\_TABLE\_RDF\_NS\_COLOR
:   HTML\_TABLE\_RDF\_NS\_COLOR in constants.php

HTML\_TABLE\_RESOURCE\_COLOR
:   HTML\_TABLE\_RESOURCE\_COLOR in constants.php

i

top

ICQ\_CHAT\_ID
:   FOAF\_RES::ICQ\_CHAT\_ID() in FOAF\_RES.php

ICQ\_CHAT\_ID
:   FOAF::ICQ\_CHAT\_ID() in FOAF\_C.php

ID
:   RDF\_RES::ID() in RDF\_RES.php

ID
:   RDF::ID() in RDF\_C.php

IDENTIFIER
:   DC\_RES::IDENTIFIER() in DC\_RES.php

IDENTIFIER
:   DC::IDENTIFIER() in DC\_C.php

IMAGE
:   DC::IMAGE() in DC\_C.php

IMAGE
:   FOAF\_RES::IMAGE() in FOAF\_RES.php

IMAGE
:   FOAF::IMAGE() in FOAF\_C.php

IMAGE
:   RSS\_RES::IMAGE() in RSS\_RES.php

IMAGE
:   RSS::IMAGE() in RSS\_C.php

IMAGE
:   DC\_RES::IMAGE() in DC\_RES.php

IMG
:   FOAF\_RES::IMG() in FOAF\_RES.php

IMG
:   FOAF::IMG() in FOAF\_C.php

IMPORTS
:   OWL::IMPORTS() in OWL\_C.php

IMPORTS
:   OWL\_RES::IMPORTS() in OWL\_RES.php

IMT
:   DC\_RES::IMT() in DC\_RES.php

IMT
:   DC::IMT() in DC\_C.php

INCOMPATIBLE\_WITH
:   OWL\_RES::INCOMPATIBLE\_WITH() in OWL\_RES.php

INCOMPATIBLE\_WITH
:   OWL::INCOMPATIBLE\_WITH() in OWL\_C.php

INDENTATION
:   INDENTATION in constants.php

INDEX\_TYPE
:   INDEX\_TYPE in constants.php

IND\_DEF
:   IND\_DEF in constants.php

IND\_SO
:   IND\_SO in constants.php

IND\_SP
:   IND\_SP in constants.php

IND\_SPO
:   IND\_SPO in constants.php

INFMODELB
:   INFMODELB in constants.php

INFMODELF
:   INFMODELF in constants.php

InfModelP.php
:   InfModelP.php in InfModelP.php

INFO
:   ATOM::INFO() in ATOM\_C.php

INFO
:   ATOM\_RES::INFO() in ATOM\_RES.php

INF\_RES\_DOMAIN
:   INF\_RES\_DOMAIN in constants.php

INF\_RES\_OWL\_INVERSEOF
:   INF\_RES\_OWL\_INVERSEOF in constants.php

INF\_RES\_OWL\_SAMEAS
:   INF\_RES\_OWL\_SAMEAS in constants.php

INF\_RES\_RANGE
:   INF\_RES\_RANGE in constants.php

INF\_RES\_RULE\_RDFS6
:   INF\_RES\_RULE\_RDFS6 in constants.php

INF\_RES\_RULE\_RDFS8
:   INF\_RES\_RULE\_RDFS8 in constants.php

INF\_RES\_RULE\_RDFS10
:   INF\_RES\_RULE\_RDFS10 in constants.php

INF\_RES\_RULE\_RDFS12
:   INF\_RES\_RULE\_RDFS12 in constants.php

INF\_RES\_RULE\_RDFS13
:   INF\_RES\_RULE\_RDFS13 in constants.php

INF\_RES\_SUBCLASSOF
:   INF\_RES\_SUBCLASSOF in constants.php

INF\_RES\_SUBPROPERTYOF
:   INF\_RES\_SUBPROPERTYOF in constants.php

INTERACTIVERESOURCE
:   DC::INTERACTIVERESOURCE() in DC\_C.php

INTERACTIVE\_RESOURCE
:   DC\_RES::INTERACTIVE\_RESOURCE() in DC\_RES.php

INTEREST
:   FOAF::INTEREST() in FOAF\_C.php

INTEREST
:   FOAF\_RES::INTEREST() in FOAF\_RES.php

INTERSECTION\_OF
:   OWL\_RES::INTERSECTION\_OF() in OWL\_RES.php

INTERSECTION\_OF
:   OWL::INTERSECTION\_OF() in OWL\_C.php

INVERSE\_FUNCTIONAL\_PROPERTY
:   OWL::INVERSE\_FUNCTIONAL\_PROPERTY() in OWL\_C.php

INVERSE\_FUNCTIONAL\_PROPERTY
:   OWL\_RES::INVERSE\_FUNCTIONAL\_PROPERTY() in OWL\_RES.php

INVERSE\_OF
:   OWL\_RES::INVERSE\_OF() in OWL\_RES.php

INVERSE\_OF
:   OWL::INVERSE\_OF() in OWL\_C.php

IN\_DESCRIPTION
:   IN\_DESCRIPTION in constants.php

IN\_PROPERTY\_EMPTY\_RESOURCE
:   IN\_PROPERTY\_EMPTY\_RESOURCE in constants.php

IN\_PROPERTY\_LITERAL
:   IN\_PROPERTY\_LITERAL in constants.php

IN\_PROPERTY\_PARSE\_TYPE\_COLLECTION
:   IN\_PROPERTY\_PARSE\_TYPE\_COLLECTION in constants.php

IN\_PROPERTY\_PARSE\_TYPE\_LITERAL
:   IN\_PROPERTY\_PARSE\_TYPE\_LITERAL in constants.php

IN\_PROPERTY\_PARSE\_TYPE\_RESOURCE
:   IN\_PROPERTY\_PARSE\_TYPE\_RESOURCE in constants.php

IN\_PROPERTY\_RESOURCE
:   IN\_PROPERTY\_RESOURCE in constants.php

IN\_PROPERTY\_UNKNOWN\_OBJECT
:   IN\_PROPERTY\_UNKNOWN\_OBJECT in constants.php

IN\_RDF
:   IN\_RDF in constants.php

IN\_TOP\_LEVEL
:   IN\_TOP\_LEVEL in constants.php

IN\_UNKNOWN
:   IN\_UNKNOWN in constants.php

IN\_XML
:   IN\_XML in constants.php

ISO639\_2
:   DC\_RES::ISO639\_2() in DC\_RES.php

ISO639\_2
:   DC::ISO639\_2() in DC\_C.php

ISO3166
:   DC::ISO3166() in DC\_C.php

ISO3166
:   DC\_RES::ISO3166() in DC\_RES.php

ISSUED
:   DC::ISSUED() in DC\_C.php

ISSUED
:   ATOM::ISSUED() in ATOM\_C.php

ISSUED
:   DC\_RES::ISSUED() in DC\_RES.php

ISSUED
:   ATOM\_RES::ISSUED() in ATOM\_RES.php

IS\_DEFINED\_BY
:   RDFS::IS\_DEFINED\_BY() in RDFS\_C.php

IS\_DEFINED\_BY
:   RDFS\_RES::IS\_DEFINED\_BY() in RDFS\_RES.php

IS\_FORMAT\_OF
:   DC::IS\_FORMAT\_OF() in DC\_C.php

IS\_FORMAT\_OF
:   DC\_RES::IS\_FORMAT\_OF() in DC\_RES.php

IS\_PART\_OF
:   DC::IS\_PART\_OF() in DC\_C.php

IS\_PART\_OF
:   DC\_RES::IS\_PART\_OF() in DC\_RES.php

IS\_REFERENCED\_BY
:   DC::IS\_REFERENCED\_BY() in DC\_C.php

IS\_REFERENCED\_BY
:   DC\_RES::IS\_REFERENCED\_BY() in DC\_RES.php

IS\_REPLACED\_BY
:   DC\_RES::IS\_REPLACED\_BY() in DC\_RES.php

IS\_REPLACED\_BY
:   DC::IS\_REPLACED\_BY() in DC\_C.php

IS\_REQUIRED\_BY
:   DC::IS\_REQUIRED\_BY() in DC\_C.php

IS\_REQUIRED\_BY
:   DC\_RES::IS\_REQUIRED\_BY() in DC\_RES.php

IS\_VERSION\_OF
:   DC\_RES::IS\_VERSION\_OF() in DC\_RES.php

IS\_VERSION\_OF
:   DC::IS\_VERSION\_OF() in DC\_C.php

ITEM
:   RSS\_RES::ITEM() in RSS\_RES.php

ITEM
:   RSS::ITEM() in RSS\_C.php

ITEMS
:   RSS::ITEMS() in RSS\_C.php

ITEMS
:   RSS\_RES::ITEMS() in RSS\_RES.php

j

top

JABBER\_ID
:   FOAF\_RES::JABBER\_ID() in FOAF\_RES.php

JABBER\_ID
:   FOAF::JABBER\_ID() in FOAF\_C.php

k

top

KEY
:   VCARD\_RES::KEY() in VCARD\_RES.php

KEY
:   VCARD::KEY() in VCARD\_C.php

KNOWS
:   FOAF\_RES::KNOWS() in FOAF\_RES.php

KNOWS
:   FOAF::KNOWS() in FOAF\_C.php

l

top

LABEL
:   RDFS\_RES::LABEL() in RDFS\_RES.php

LABEL
:   VCARD\_RES::LABEL() in VCARD\_RES.php

LABEL
:   RDFS::LABEL() in RDFS\_C.php

LABEL
:   VCARD::LABEL() in VCARD\_C.php

LANG
:   ATOM\_RES::LANG() in ATOM\_RES.php

LANG
:   ATOM::LANG() in ATOM\_C.php

LANGUAGE
:   DC::LANGUAGE() in DC\_C.php

LANGUAGE
:   DC\_RES::LANGUAGE() in DC\_RES.php

LCC
:   DC\_RES::LCC() in DC\_RES.php

LCC
:   DC::LCC() in DC\_C.php

LCSH
:   DC::LCSH() in DC\_C.php

LCSH
:   DC\_RES::LCSH() in DC\_RES.php

LI
:   RDF\_RES::LI() in RDF\_RES.php

LI
:   RDF::LI() in RDF\_C.php

LICENSE
:   DC\_RES::LICENSE() in DC\_RES.php

LICENSE
:   DC::LICENSE() in DC\_C.php

LINEFEED
:   LINEFEED in constants.php

LINK
:   RSS::LINK() in RSS\_C.php

LINK
:   ATOM\_RES::LINK() in ATOM\_RES.php

LINK
:   RSS\_RES::LINK() in RSS\_RES.php

LINK
:   ATOM::LINK() in ATOM\_C.php

LITERAL
:   RDF\_RES::LITERAL() in RDF\_RES.php

LITERAL
:   RDF::LITERAL() in RDF\_C.php

LITERAL
:   RDFS::LITERAL() in RDFS\_C.php

LITERAL
:   RDFS\_RES::LITERAL() in RDFS\_RES.php

LOCALITY
:   VCARD::LOCALITY() in VCARD\_C.php

LOCALITY
:   VCARD\_RES::LOCALITY() in VCARD\_RES.php

LOGO
:   FOAF\_RES::LOGO() in FOAF\_RES.php

LOGO
:   FOAF::LOGO() in FOAF\_C.php

m

top

MADE
:   FOAF\_RES::MADE() in FOAF\_RES.php

MADE
:   FOAF::MADE() in FOAF\_C.php

MAILER
:   VCARD::MAILER() in VCARD\_C.php

MAILER
:   VCARD\_RES::MAILER() in VCARD\_RES.php

MAKER
:   FOAF\_RES::MAKER() in FOAF\_RES.php

MAKER
:   FOAF::MAKER() in FOAF\_C.php

MAX\_ALLOWED\_ABBREVIATED\_LENGTH
:   MAX\_ALLOWED\_ABBREVIATED\_LENGTH in constants.php

MAX\_CARDINALITY
:   OWL\_RES::MAX\_CARDINALITY() in OWL\_RES.php

MAX\_CARDINALITY
:   OWL::MAX\_CARDINALITY() in OWL\_C.php

MBOX
:   FOAF::MBOX() in FOAF\_C.php

MBOX
:   FOAF\_RES::MBOX() in FOAF\_RES.php

MBOX\_SHA1SUM
:   FOAF\_RES::MBOX\_SHA1SUM() in FOAF\_RES.php

MBOX\_SHA1SUM
:   FOAF::MBOX\_SHA1SUM() in FOAF\_C.php

MEDIATOR
:   DC\_RES::MEDIATOR() in DC\_RES.php

MEDIATOR
:   DC::MEDIATOR() in DC\_C.php

MEDIUM
:   DC::MEDIUM() in DC\_C.php

MEDIUM
:   DC\_RES::MEDIUM() in DC\_RES.php

MEMBER
:   FOAF\_RES::MEMBER() in FOAF\_RES.php

MEMBER
:   RDFS\_RES::MEMBER() in RDFS\_RES.php

MEMBER
:   RDFS::MEMBER() in RDFS\_C.php

MEMBER
:   FOAF::MEMBER() in FOAF\_C.php

MEMBERSHIP\_CLASS
:   FOAF\_RES::MEMBERSHIP\_CLASS() in FOAF\_RES.php

MEMBERSHIP\_CLASS
:   FOAF::MEMBERSHIP\_CLASS() in FOAF\_C.php

MEMMODEL
:   MEMMODEL in constants.php

MESH
:   DC::MESH() in DC\_C.php

MESH
:   DC\_RES::MESH() in DC\_RES.php

MIN\_CARDINALITY
:   OWL::MIN\_CARDINALITY() in OWL\_C.php

MIN\_CARDINALITY
:   OWL\_RES::MIN\_CARDINALITY() in OWL\_RES.php

MODE
:   ATOM\_RES::MODE() in ATOM\_RES.php

MODE
:   ATOM::MODE() in ATOM\_C.php

ModelP.php
:   ModelP.php in ModelP.php

MODIFIED
:   DC::MODIFIED() in DC\_C.php

MODIFIED
:   ATOM::MODIFIED() in ATOM\_C.php

MODIFIED
:   DC\_RES::MODIFIED() in DC\_RES.php

MODIFIED
:   ATOM\_RES::MODIFIED() in ATOM\_RES.php

MOVINGIMAGE
:   DC::MOVINGIMAGE() in DC\_C.php

MOVINGIMAGE
:   DC\_RES::MOVINGIMAGE() in DC\_RES.php

MSN\_CHAT\_ID
:   FOAF\_RES::MSN\_CHAT\_ID() in FOAF\_RES.php

MSN\_CHAT\_ID
:   FOAF::MSN\_CHAT\_ID() in FOAF\_C.php

MYERS\_BRIGGS
:   FOAF::MYERS\_BRIGGS() in FOAF\_C.php

MYERS\_BRIGGS
:   FOAF\_RES::MYERS\_BRIGGS() in FOAF\_RES.php

n

top

N
:   VCARD\_RES::N() in VCARD\_RES.php

N
:   VCARD::N() in VCARD\_C.php

N3SER\_BNODE\_SHORT
:   N3SER\_BNODE\_SHORT in constants.php

NAME
:   VCARD\_RES::NAME() in VCARD\_RES.php

NAME
:   RSS::NAME() in RSS\_C.php

NAME
:   VCARD::NAME() in VCARD\_C.php

NAME
:   RSS\_RES::NAME() in RSS\_RES.php

NAME
:   ATOM::NAME() in ATOM\_C.php

NAME
:   FOAF\_RES::NAME() in FOAF\_RES.php

NAME
:   ATOM\_RES::NAME() in ATOM\_RES.php

NAME
:   FOAF::NAME() in FOAF\_C.php

NAMESPACE\_SEPARATOR\_CHAR
:   NAMESPACE\_SEPARATOR\_CHAR in constants.php

NAMESPACE\_SEPARATOR\_STRING
:   NAMESPACE\_SEPARATOR\_STRING in constants.php

NICK
:   FOAF\_RES::NICK() in FOAF\_RES.php

NICK
:   FOAF::NICK() in FOAF\_C.php

NICKNAME
:   VCARD\_RES::NICKNAME() in VCARD\_RES.php

NICKNAME
:   VCARD::NICKNAME() in VCARD\_C.php

NIL
:   RDF::NIL() in RDF\_C.php

NIL
:   RDF\_RES::NIL() in RDF\_RES.php

NODE\_ID
:   RDF::NODE\_ID() in RDF\_C.php

NODE\_ID
:   RDF\_RES::NODE\_ID() in RDF\_RES.php

NOTHING
:   OWL::NOTHING() in OWL\_C.php

NOTHING
:   OWL\_RES::NOTHING() in OWL\_RES.php

NO\_INDEX
:   NO\_INDEX in constants.php

NPROPERTIES
:   VCARD\_RES::NPROPERTIES() in VCARD\_RES.php

NPROPERTIES
:   VCARD::NPROPERTIES() in VCARD\_C.php

o

top

OBJECT
:   RDF\_RES::OBJECT() in RDF\_RES.php

OBJECT
:   RDF::OBJECT() in RDF\_C.php

OBJECT\_CLASS
:   OWL::OBJECT\_CLASS() in OWL\_C.php

OBJECT\_CLASS
:   OWL\_RES::OBJECT\_CLASS() in OWL\_RES.php

OBJECT\_PROPERTY
:   OWL\_RES::OBJECT\_PROPERTY() in OWL\_RES.php

OBJECT\_PROPERTY
:   OWL::OBJECT\_PROPERTY() in OWL\_C.php

OBJECT\_RESTRICTION
:   OWL::OBJECT\_RESTRICTION() in OWL\_C.php

OBJECT\_RESTRICTION
:   OWL\_RES::OBJECT\_RESTRICTION() in OWL\_RES.php

ONE\_OF
:   OWL\_RES::ONE\_OF() in OWL\_RES.php

ONE\_OF
:   OWL::ONE\_OF() in OWL\_C.php

ONLINE\_ACCOUNT
:   FOAF\_RES::ONLINE\_ACCOUNT() in FOAF\_RES.php

ONLINE\_ACCOUNT
:   FOAF::ONLINE\_ACCOUNT() in FOAF\_C.php

ONLINE\_CHAT\_ACCOUNT
:   FOAF\_RES::ONLINE\_CHAT\_ACCOUNT() in FOAF\_RES.php

ONLINE\_CHAT\_ACCOUNT
:   FOAF::ONLINE\_CHAT\_ACCOUNT() in FOAF\_C.php

ONLINE\_ECOMMERCE\_ACCOUNT
:   FOAF::ONLINE\_ECOMMERCE\_ACCOUNT() in FOAF\_C.php

ONLINE\_ECOMMERCE\_ACCOUNT
:   FOAF\_RES::ONLINE\_ECOMMERCE\_ACCOUNT() in FOAF\_RES.php

ONLINE\_GAMING\_ACCOUNT
:   FOAF::ONLINE\_GAMING\_ACCOUNT() in FOAF\_C.php

ONLINE\_GAMING\_ACCOUNT
:   FOAF\_RES::ONLINE\_GAMING\_ACCOUNT() in FOAF\_RES.php

ONTMODEL
:   ONTMODEL in constants.php

ONTOLOGY
:   OWL\_RES::ONTOLOGY() in OWL\_RES.php

ONTOLOGY
:   OWL::ONTOLOGY() in OWL\_C.php

ON\_PROPERTY
:   OWL\_RES::ON\_PROPERTY() in OWL\_RES.php

ON\_PROPERTY
:   OWL::ON\_PROPERTY() in OWL\_C.php

ORGANIZATION
:   FOAF\_RES::ORGANIZATION() in FOAF\_RES.php

ORGANIZATION
:   FOAF::ORGANIZATION() in FOAF\_C.php

ORGNAME
:   VCARD::ORGNAME() in VCARD\_C.php

ORGNAME
:   VCARD\_RES::ORGNAME() in VCARD\_RES.php

ORGPROPERTIES
:   VCARD::ORGPROPERTIES() in VCARD\_C.php

ORGPROPERTIES
:   VCARD\_RES::ORGPROPERTIES() in VCARD\_RES.php

ORGUNIT
:   VCARD::ORGUNIT() in VCARD\_C.php

ORGUNIT
:   VCARD\_RES::ORGUNIT() in VCARD\_RES.php

OTHER
:   VCARD\_RES::OTHER() in VCARD\_RES.php

OTHER
:   VCARD::OTHER() in VCARD\_C.php

OWL
:   OWL in OWL\_C.php

OWL\_CLASS
:   OWL::OWL\_CLASS() in OWL\_C.php

OWL\_CLASS
:   OWL\_RES::OWL\_CLASS() in OWL\_RES.php

OWL\_INVERSE\_OF
:   OWL\_INVERSE\_OF in constants.php

OWL\_NS
:   OWL\_NS in constants.php

OWL\_PREFIX
:   OWL\_PREFIX in constants.php

OWL\_RES
:   OWL\_RES in OWL\_RES.php

OWL\_SAME\_AS
:   OWL\_SAME\_AS in constants.php

OWL\_URI
:   OWL\_URI in constants.php

OWL\_C.php
:   OWL\_C.php in OWL\_C.php

OWL.php
:   OWL.php in OWL.php

OWL\_RES.php
:   OWL\_RES.php in OWL\_RES.php

p

top

PACKAGE\_DBASE
:   PACKAGE\_DBASE in constants.php

PACKAGE\_INFMODEL
:   PACKAGE\_INFMODEL in constants.php

PACKAGE\_MODEL
:   PACKAGE\_MODEL in constants.php

PACKAGE\_ONTMODEL
:   PACKAGE\_ONTMODEL in constants.php

PACKAGE\_RDQL
:   PACKAGE\_RDQL in constants.php

PACKAGE\_RESMODEL
:   PACKAGE\_RESMODEL in constants.php

PACKAGE\_SYNTAX\_GRDDL
:   PACKAGE\_SYNTAX\_GRDDL in constants.php

PACKAGE\_SYNTAX\_N3
:   PACKAGE\_SYNTAX\_N3 in constants.php

PACKAGE\_SYNTAX\_RDF
:   PACKAGE\_SYNTAX\_RDF in constants.php

PACKAGE\_UTILITY
:   PACKAGE\_UTILITY in constants.php

PACKAGE\_VOCABULARY
:   PACKAGE\_VOCABULARY in constants.php

PAGE
:   FOAF::PAGE() in FOAF\_C.php

PAGE
:   FOAF\_RES::PAGE() in FOAF\_RES.php

PARSE\_TYPE
:   RDF::PARSE\_TYPE() in RDF\_C.php

PARSE\_TYPE
:   RDF\_RES::PARSE\_TYPE() in RDF\_RES.php

PARSE\_TYPE\_RESOURCE
:   RDF::PARSE\_TYPE\_RESOURCE() in RDF\_C.php

PARSE\_TYPE\_RESOURCE
:   RDF\_RES::PARSE\_TYPE\_RESOURCE() in RDF\_RES.php

PAST\_PROJECT
:   FOAF::PAST\_PROJECT() in FOAF\_C.php

PAST\_PROJECT
:   FOAF\_RES::PAST\_PROJECT() in FOAF\_RES.php

PCODE
:   VCARD::PCODE() in VCARD\_C.php

PCODE
:   VCARD\_RES::PCODE() in VCARD\_RES.php

PERIOD
:   DC::PERIOD() in DC\_C.php

PERIOD
:   DC\_RES::PERIOD() in DC\_RES.php

PERSON
:   ATOM\_RES::PERSON() in ATOM\_RES.php

PERSON
:   ATOM::PERSON() in ATOM\_C.php

PERSON
:   FOAF\_RES::PERSON() in FOAF\_RES.php

PERSON
:   FOAF::PERSON() in FOAF\_C.php

PERSONAL\_PROFILE\_DOCUMENT
:   FOAF\_RES::PERSONAL\_PROFILE\_DOCUMENT() in FOAF\_RES.php

PERSONAL\_PROFILE\_DOCUMENT
:   FOAF::PERSONAL\_PROFILE\_DOCUMENT() in FOAF\_C.php

PHONE
:   FOAF\_RES::PHONE() in FOAF\_RES.php

PHONE
:   FOAF::PHONE() in FOAF\_C.php

PHOTO
:   VCARD::PHOTO() in VCARD\_C.php

PHOTO
:   VCARD\_RES::PHOTO() in VCARD\_RES.php

PHYSICALOBJECT
:   DC\_RES::PHYSICALOBJECT() in DC\_RES.php

PHYSICALOBJECT
:   DC::PHYSICALOBJECT() in DC\_C.php

PLAN
:   FOAF\_RES::PLAN() in FOAF\_RES.php

PLAN
:   FOAF::PLAN() in FOAF\_C.php

POBOX
:   VCARD\_RES::POBOX() in VCARD\_RES.php

POBOX
:   VCARD::POBOX() in VCARD\_C.php

POINT
:   DC\_RES::POINT() in DC\_RES.php

POINT
:   DC::POINT() in DC\_C.php

PREDICATE
:   RDF::PREDICATE() in RDF\_C.php

PREDICATE
:   RDF\_RES::PREDICATE() in RDF\_RES.php

PREFIX
:   VCARD::PREFIX() in VCARD\_C.php

PREFIX
:   VCARD\_RES::PREFIX() in VCARD\_RES.php

PRIMARY\_TOPIC
:   FOAF\_RES::PRIMARY\_TOPIC() in FOAF\_RES.php

PRIMARY\_TOPIC
:   FOAF::PRIMARY\_TOPIC() in FOAF\_C.php

PRIOR\_VERSION
:   OWL::PRIOR\_VERSION() in OWL\_C.php

PRIOR\_VERSION
:   OWL\_RES::PRIOR\_VERSION() in OWL\_RES.php

PRODID
:   VCARD::PRODID() in VCARD\_C.php

PRODID
:   VCARD\_RES::PRODID() in VCARD\_RES.php

PROJECT
:   FOAF::PROJECT() in FOAF\_C.php

PROJECT
:   FOAF\_RES::PROJECT() in FOAF\_RES.php

PROPERTY
:   OWL::PROPERTY() in OWL\_C.php

PROPERTY
:   OWL\_RES::PROPERTY() in OWL\_RES.php

PROPERTY
:   RDF::PROPERTY() in RDF\_C.php

PROPERTY
:   RDF\_RES::PROPERTY() in RDF\_RES.php

PUBLICATIONS
:   FOAF\_RES::PUBLICATIONS() in FOAF\_RES.php

PUBLICATIONS
:   FOAF::PUBLICATIONS() in FOAF\_C.php

PUBLISHER
:   DC\_RES::PUBLISHER() in DC\_RES.php

PUBLISHER
:   DC::PUBLISHER() in DC\_C.php

r

top

RANGE
:   RDFS::RANGE() in RDFS\_C.php

RANGE
:   RDFS\_RES::RANGE() in RDFS\_RES.php

RDF
:   RDF in RDF\_C.php

RDFAPI\_ERROR
:   RDFAPI\_ERROR in constants.php

RdfAPI.php
:   RdfAPI.php in RdfAPI.php

RDFS
:   RDFS in RDFS\_C.php

RDFS\_CLASS
:   RDFS::RDFS\_CLASS() in RDFS\_C.php

RDFS\_CLASS
:   RDFS\_RES::RDFS\_CLASS() in RDFS\_RES.php

RDFS\_CLASS
:   RDFS\_CLASS in constants.php

RDFS\_COMMENT
:   RDFS\_COMMENT in constants.php

RDFS\_DATATYPE
:   RDFS\_DATATYPE in constants.php

RDFS\_DOMAIN
:   RDFS\_DOMAIN in constants.php

RDFS\_IS\_DEFINED\_BY
:   RDFS\_IS\_DEFINED\_BY in constants.php

RDFS\_LABEL
:   RDFS\_LABEL in constants.php

RDFS\_LITERAL
:   RDFS\_LITERAL in constants.php

RDFS\_RANGE
:   RDFS\_RANGE in constants.php

RDFS\_RES
:   RDFS\_RES in RDFS\_RES.php

RDFS\_RESOURCE
:   RDFS\_RESOURCE in constants.php

RDFS\_SEE\_ALSO
:   RDFS\_SEE\_ALSO in constants.php

RDFS\_SUBCLASSOF
:   RDFS\_SUBCLASSOF in constants.php

RDFS\_SUBPROPERTYOF
:   RDFS\_SUBPROPERTYOF in constants.php

RDFS\_VOCABULARY
:   RDFS\_VOCABULARY in constants.php

RDF\_ABOUT
:   RDF\_ABOUT in constants.php

RDF\_ABOUT\_EACH
:   RDF\_ABOUT\_EACH in constants.php

RDF\_ABOUT\_EACH\_PREFIX
:   RDF\_ABOUT\_EACH\_PREFIX in constants.php

RDF\_ALT
:   RDF\_ALT in constants.php

RDF\_BAG
:   RDF\_BAG in constants.php

RDF\_BAG\_ID
:   RDF\_BAG\_ID in constants.php

RDF\_DATATYPE
:   RDF\_DATATYPE in constants.php

RDF\_DATATYPE\_SCHEMA\_URI
:   RDF\_DATATYPE\_SCHEMA\_URI in constants.php

RDF\_DESCRIPTION
:   RDF\_DESCRIPTION in constants.php

RDF\_FIRST
:   RDF\_FIRST in constants.php

RDF\_ID
:   RDF\_ID in constants.php

RDF\_LI
:   RDF\_LI in constants.php

RDF\_LIST
:   RDF::RDF\_LIST() in RDF\_C.php

RDF\_LIST
:   RDF\_LIST in constants.php

RDF\_LIST
:   RDF\_RES::RDF\_LIST() in RDF\_RES.php

RDF\_NAMESPACE\_PREFIX
:   RDF\_NAMESPACE\_PREFIX in constants.php

RDF\_NAMESPACE\_URI
:   RDF\_NAMESPACE\_URI in constants.php

RDF\_NIL
:   RDF\_NIL in constants.php

RDF\_NODEID
:   RDF\_NODEID in constants.php

RDF\_OBJECT
:   RDF\_OBJECT in constants.php

RDF\_OBJECT\_TYPE\_BNODE
:   RDF\_OBJECT\_TYPE\_BNODE in constants.php

RDF\_OBJECT\_TYPE\_LITERAL
:   RDF\_OBJECT\_TYPE\_LITERAL in constants.php

RDF\_OBJECT\_TYPE\_RESOURCE
:   RDF\_OBJECT\_TYPE\_RESOURCE in constants.php

RDF\_OBJECT\_TYPE\_XML
:   RDF\_OBJECT\_TYPE\_XML in constants.php

RDF\_PARSE\_TYPE
:   RDF\_PARSE\_TYPE in constants.php

RDF\_PARSE\_TYPE\_COLLECTION
:   RDF\_PARSE\_TYPE\_COLLECTION in constants.php

RDF\_PARSE\_TYPE\_LITERAL
:   RDF\_PARSE\_TYPE\_LITERAL in constants.php

RDF\_PARSE\_TYPE\_RESOURCE
:   RDF\_PARSE\_TYPE\_RESOURCE in constants.php

RDF\_PREDICATE
:   RDF\_PREDICATE in constants.php

RDF\_PROPERTY
:   RDF\_PROPERTY in constants.php

RDF\_RDF
:   RDF\_RDF in constants.php

RDF\_RES
:   RDF\_RES in RDF\_RES.php

RDF\_RESOURCE
:   RDF\_RESOURCE in constants.php

RDF\_REST
:   RDF\_REST in constants.php

RDF\_SCHEMA\_PREFIX
:   RDF\_SCHEMA\_PREFIX in constants.php

RDF\_SCHEMA\_URI
:   RDF\_SCHEMA\_URI in constants.php

RDF\_SEEALSO
:   RDF\_SEEALSO in constants.php

RDF\_SEQ
:   RDF\_SEQ in constants.php

RDF\_STATEMENT
:   RDF\_STATEMENT in constants.php

RDF\_SUBJECT
:   RDF\_SUBJECT in constants.php

RDF\_SUBJECT\_TYPE\_ANONYMOUS
:   RDF\_SUBJECT\_TYPE\_ANONYMOUS in constants.php

RDF\_SUBJECT\_TYPE\_BNODE
:   RDF\_SUBJECT\_TYPE\_BNODE in constants.php

RDF\_SUBJECT\_TYPE\_DISTRIBUTED
:   RDF\_SUBJECT\_TYPE\_DISTRIBUTED in constants.php

RDF\_SUBJECT\_TYPE\_PREFIX
:   RDF\_SUBJECT\_TYPE\_PREFIX in constants.php

RDF\_SUBJECT\_TYPE\_URI
:   RDF\_SUBJECT\_TYPE\_URI in constants.php

RDF\_TYPE
:   RDF\_TYPE in constants.php

RDF\_VALUE
:   RDF\_VALUE in constants.php

RDF\_XMLLITERAL
:   RDF\_XMLLITERAL in constants.php

RDQL\_AND\_ERR
:   RDQL\_AND\_ERR in constants.php

RDQL\_ERR
:   RDQL\_ERR in constants.php

RDQL.php
:   RDQL.php in RDQL.php

RDQL\_SEL\_ERR
:   RDQL\_SEL\_ERR in constants.php

RDQL\_SRC\_ERR
:   RDQL\_SRC\_ERR in constants.php

RDQL\_SYN\_ERR
:   RDQL\_SYN\_ERR in constants.php

RDQL\_USG\_ERR
:   RDQL\_USG\_ERR in constants.php

RDQL\_WHR\_ERR
:   RDQL\_WHR\_ERR in constants.php

REFERENCES
:   DC::REFERENCES() in DC\_C.php

REFERENCES
:   DC\_RES::REFERENCES() in DC\_RES.php

REGION
:   VCARD\_RES::REGION() in VCARD\_RES.php

REGION
:   VCARD::REGION() in VCARD\_C.php

REL
:   ATOM::REL() in ATOM\_C.php

REL
:   ATOM\_RES::REL() in ATOM\_RES.php

REPLACES
:   DC::REPLACES() in DC\_C.php

REPLACES
:   DC\_RES::REPLACES() in DC\_RES.php

REQUIRES
:   DC::REQUIRES() in DC\_C.php

REQUIRES
:   DC\_RES::REQUIRES() in DC\_RES.php

RESMODEL
:   RESMODEL in constants.php

ResModelP.php
:   ResModelP.php in ResModelP.php

RESOURCE
:   RDFS\_RES::RESOURCE() in RDFS\_RES.php

RESOURCE
:   RDF::RESOURCE() in RDF\_C.php

RESOURCE
:   RDF\_RES::RESOURCE() in RDF\_RES.php

RESOURCE
:   RDFS::RESOURCE() in RDFS\_C.php

REST
:   RDF::REST() in RDF\_C.php

REST
:   RDF\_RES::REST() in RDF\_RES.php

RESTRICTION
:   OWL::RESTRICTION() in OWL\_C.php

RESTRICTION
:   OWL\_RES::RESTRICTION() in OWL\_RES.php

REV
:   VCARD\_RES::REV() in VCARD\_RES.php

REV
:   VCARD::REV() in VCARD\_C.php

RFC1766
:   DC\_RES::RFC1766() in DC\_RES.php

RFC1766
:   DC::RFC1766() in DC\_C.php

RFC3066
:   DC\_RES::RFC3066() in DC\_RES.php

RFC3066
:   DC::RFC3066() in DC\_C.php

RIGHTS
:   DC\_RES::RIGHTS() in DC\_RES.php

RIGHTS
:   DC::RIGHTS() in DC\_C.php

RIGHTS\_HOLDER
:   DC\_RES::RIGHTS\_HOLDER() in DC\_RES.php

RIGHTS\_HOLDER
:   DC::RIGHTS\_HOLDER() in DC\_C.php

ROLE
:   VCARD\_RES::ROLE() in VCARD\_RES.php

ROLE
:   VCARD::ROLE() in VCARD\_C.php

RSS
:   RSS in RSS\_C.php

RSS\_NS
:   RSS\_NS in constants.php

RSS\_RES
:   RSS\_RES in RSS\_RES.php

RDFS\_C.php
:   RDFS\_C.php in RDFS\_C.php

RDFS.php
:   RDFS.php in RDFS.php

RDFS\_RES.php
:   RDFS\_RES.php in RDFS\_RES.php

RDF\_C.php
:   RDF\_C.php in RDF\_C.php

RDF.php
:   RDF.php in RDF.php

RDF\_RES.php
:   RDF\_RES.php in RDF\_RES.php

RSS\_C.php
:   RSS\_C.php in RSS\_C.php

RSS.php
:   RSS.php in RSS.php

RSS\_RES.php
:   RSS\_RES.php in RSS\_RES.php

s

top

SAME\_AS
:   OWL::SAME\_AS() in OWL\_C.php

SAME\_AS
:   OWL\_RES::SAME\_AS() in OWL\_RES.php

SAME\_CLASS\_AS
:   OWL::SAME\_CLASS\_AS() in OWL\_C.php

SAME\_CLASS\_AS
:   OWL\_RES::SAME\_CLASS\_AS() in OWL\_RES.php

SAME\_INDIVIDUAL\_AS
:   OWL\_RES::SAME\_INDIVIDUAL\_AS() in OWL\_RES.php

SAME\_INDIVIDUAL\_AS
:   OWL::SAME\_INDIVIDUAL\_AS() in OWL\_C.php

SAME\_PROPERTY\_AS
:   OWL\_RES::SAME\_PROPERTY\_AS() in OWL\_RES.php

SAME\_PROPERTY\_AS
:   OWL::SAME\_PROPERTY\_AS() in OWL\_C.php

SCHOOL\_HOMEPAGE
:   FOAF::SCHOOL\_HOMEPAGE() in FOAF\_C.php

SCHOOL\_HOMEPAGE
:   FOAF\_RES::SCHOOL\_HOMEPAGE() in FOAF\_RES.php

SEEALSO
:   RDFS::SEEALSO() in RDFS\_C.php

SEEALSO
:   RDFS\_RES::SEEALSO() in RDFS\_RES.php

SEE\_ALSO
:   RDF::SEE\_ALSO() in RDF\_C.php

SEE\_ALSO
:   RDF\_RES::SEE\_ALSO() in RDF\_RES.php

SEQ
:   RDF\_RES::SEQ() in RDF\_RES.php

SEQ
:   RDF::SEQ() in RDF\_C.php

SERVICE
:   DC::SERVICE() in DC\_C.php

SERVICE
:   DC\_RES::SERVICE() in DC\_RES.php

SER\_RDF\_QNAMES
:   SER\_RDF\_QNAMES in constants.php

SER\_SORT\_MODEL
:   SER\_SORT\_MODEL in constants.php

SER\_USE\_ATTRIBUTES
:   SER\_USE\_ATTRIBUTES in constants.php

SER\_USE\_ENTITIES
:   SER\_USE\_ENTITIES in constants.php

SER\_XML\_DECLARATION
:   SER\_XML\_DECLARATION in constants.php

SHA1
:   FOAF::SHA1() in FOAF\_C.php

SHA1
:   FOAF\_RES::SHA1() in FOAF\_RES.php

SOFTWARE
:   DC\_RES::SOFTWARE() in DC\_RES.php

SOFTWARE
:   DC::SOFTWARE() in DC\_C.php

SOME\_VALUES\_FROM
:   OWL::SOME\_VALUES\_FROM() in OWL\_C.php

SOME\_VALUES\_FROM
:   OWL\_RES::SOME\_VALUES\_FROM() in OWL\_RES.php

SORT\_STRING
:   VCARD::SORT\_STRING() in VCARD\_C.php

SORT\_STRING
:   VCARD\_RES::SORT\_STRING() in VCARD\_RES.php

SOUND
:   DC::SOUND() in DC\_C.php

SOUND
:   VCARD\_RES::SOUND() in VCARD\_RES.php

SOUND
:   DC\_RES::SOUND() in DC\_RES.php

SOUND
:   VCARD::SOUND() in VCARD\_C.php

SOURCE
:   VCARD\_RES::SOURCE() in VCARD\_RES.php

SOURCE
:   DC::SOURCE() in DC\_C.php

SOURCE
:   DC\_RES::SOURCE() in DC\_RES.php

SOURCE
:   VCARD::SOURCE() in VCARD\_C.php

SPATIAL
:   DC::SPATIAL() in DC\_C.php

SPATIAL
:   DC\_RES::SPATIAL() in DC\_RES.php

STATEMENT
:   RDF::STATEMENT() in RDF\_C.php

STATEMENT
:   RDF\_RES::STATEMENT() in RDF\_RES.php

STILLIMAGE
:   DC::STILLIMAGE() in DC\_C.php

STILLIMAGE
:   DC\_RES::STILLIMAGE() in DC\_RES.php

STREET
:   VCARD\_RES::STREET() in VCARD\_RES.php

STREET
:   VCARD::STREET() in VCARD\_C.php

SUBJECT
:   RDF::SUBJECT() in RDF\_C.php

SUBJECT
:   DC::SUBJECT() in DC\_C.php

SUBJECT
:   DC\_RES::SUBJECT() in DC\_RES.php

SUBJECT
:   RDF\_RES::SUBJECT() in RDF\_RES.php

SUB\_CLASS\_OF
:   RDFS::SUB\_CLASS\_OF() in RDFS\_C.php

SUB\_CLASS\_OF
:   RDFS\_RES::SUB\_CLASS\_OF() in RDFS\_RES.php

SUB\_PROPERTY\_OF
:   RDFS\_RES::SUB\_PROPERTY\_OF() in RDFS\_RES.php

SUB\_PROPERTY\_OF
:   RDFS::SUB\_PROPERTY\_OF() in RDFS\_C.php

SUFFIX
:   VCARD::SUFFIX() in VCARD\_C.php

SUFFIX
:   VCARD\_RES::SUFFIX() in VCARD\_RES.php

SUMMARY
:   ATOM\_RES::SUMMARY() in ATOM\_RES.php

SUMMARY
:   ATOM::SUMMARY() in ATOM\_C.php

SURNAME
:   FOAF::SURNAME() in FOAF\_C.php

SURNAME
:   FOAF\_RES::SURNAME() in FOAF\_RES.php

SYMMETRIC\_PROPERTY
:   OWL::SYMMETRIC\_PROPERTY() in OWL\_C.php

SYMMETRIC\_PROPERTY
:   OWL\_RES::SYMMETRIC\_PROPERTY() in OWL\_RES.php

SyntaxGRDDL.php
:   SyntaxGRDDL.php in SyntaxGRDDL.php

SyntaxN3.php
:   SyntaxN3.php in SyntaxN3.php

SyntaxRDF.php
:   SyntaxRDF.php in SyntaxRDF.php

t

top

TABLE\_OF\_CONTENTS
:   DC::TABLE\_OF\_CONTENTS() in DC\_C.php

TABLE\_OF\_CONTENTS
:   DC\_RES::TABLE\_OF\_CONTENTS() in DC\_RES.php

TAGLINE
:   ATOM\_RES::TAGLINE() in ATOM\_RES.php

TAGLINE
:   ATOM::TAGLINE() in ATOM\_C.php

TEL
:   VCARD::TEL() in VCARD\_C.php

TEL
:   VCARD\_RES::TEL() in VCARD\_RES.php

TELTYPES
:   VCARD\_RES::TELTYPES() in VCARD\_RES.php

TELTYPES
:   VCARD::TELTYPES() in VCARD\_C.php

TEMPORAL
:   DC\_RES::TEMPORAL() in DC\_RES.php

TEMPORAL
:   DC::TEMPORAL() in DC\_C.php

TEXT
:   DC\_RES::TEXT() in DC\_RES.php

TEXT
:   DC::TEXT() in DC\_C.php

TEXTINPUT
:   RSS::TEXTINPUT() in RSS\_C.php

TEXTINPUT
:   RSS\_RES::TEXTINPUT() in RSS\_RES.php

TGN
:   DC\_RES::TGN() in DC\_RES.php

TGN
:   DC::TGN() in DC\_C.php

THEME
:   FOAF::THEME() in FOAF\_C.php

THEME
:   FOAF\_RES::THEME() in FOAF\_RES.php

THING
:   OWL\_RES::THING() in OWL\_RES.php

THING
:   OWL::THING() in OWL\_C.php

THUMBNAIL
:   FOAF::THUMBNAIL() in FOAF\_C.php

THUMBNAIL
:   FOAF\_RES::THUMBNAIL() in FOAF\_RES.php

TIPJAR
:   FOAF::TIPJAR() in FOAF\_C.php

TIPJAR
:   FOAF\_RES::TIPJAR() in FOAF\_RES.php

TITLE
:   VCARD\_RES::TITLE() in VCARD\_RES.php

TITLE
:   ATOM::TITLE() in ATOM\_C.php

TITLE
:   ATOM\_RES::TITLE() in ATOM\_RES.php

TITLE
:   RSS::TITLE() in RSS\_C.php

TITLE
:   RSS\_RES::TITLE() in RSS\_RES.php

TITLE
:   VCARD::TITLE() in VCARD\_C.php

TITLE
:   FOAF\_RES::TITLE() in FOAF\_RES.php

TITLE
:   FOAF::TITLE() in FOAF\_C.php

TITLE
:   DC::TITLE() in DC\_C.php

TITLE
:   DC\_RES::TITLE() in DC\_RES.php

TOPIC
:   FOAF\_RES::TOPIC() in FOAF\_RES.php

TOPIC
:   FOAF::TOPIC() in FOAF\_C.php

TOPIC\_INTEREST
:   FOAF::TOPIC\_INTEREST() in FOAF\_C.php

TOPIC\_INTEREST
:   FOAF\_RES::TOPIC\_INTEREST() in FOAF\_RES.php

TRANSITIVE\_PROPERTY
:   OWL\_RES::TRANSITIVE\_PROPERTY() in OWL\_RES.php

TRANSITIVE\_PROPERTY
:   OWL::TRANSITIVE\_PROPERTY() in OWL\_C.php

TYPE
:   ATOM::TYPE() in ATOM\_C.php

TYPE
:   DC\_RES::TYPE() in DC\_RES.php

TYPE
:   DC::TYPE() in DC\_C.php

TYPE
:   RDF::TYPE() in RDF\_C.php

TYPE
:   ATOM\_RES::TYPE() in ATOM\_RES.php

TYPE
:   RDF\_RES::TYPE() in RDF\_RES.php

TZ
:   VCARD\_RES::TZ() in VCARD\_RES.php

TZ
:   VCARD::TZ() in VCARD\_C.php

TZTYPES
:   VCARD\_RES::TZTYPES() in VCARD\_RES.php

TZTYPES
:   VCARD::TZTYPES() in VCARD\_C.php

u

top

UDC
:   DC::UDC() in DC\_C.php

UDC
:   DC\_RES::UDC() in DC\_RES.php

UID
:   VCARD\_RES::UID() in VCARD\_RES.php

UID
:   VCARD::UID() in VCARD\_C.php

UNIC\_RDF
:   UNIC\_RDF in constants.php

UNION\_OF
:   OWL::UNION\_OF() in OWL\_C.php

UNION\_OF
:   OWL\_RES::UNION\_OF() in OWL\_RES.php

URI
:   DC\_RES::URI() in DC\_RES.php

URI
:   DC::URI() in DC\_C.php

URL
:   RSS\_RES::URL() in RSS\_RES.php

URL
:   ATOM::URL() in ATOM\_C.php

URL
:   RSS::URL() in RSS\_C.php

URL
:   ATOM\_RES::URL() in ATOM\_RES.php

USE\_ANY\_QUOTE
:   USE\_ANY\_QUOTE in constants.php

USE\_CDATA
:   USE\_CDATA in constants.php

Utility.php
:   Utility.php in Utility.php

v

top

VALID
:   DC::VALID() in DC\_C.php

VALID
:   DC\_RES::VALID() in DC\_RES.php

VALIDATE\_IDS
:   VALIDATE\_IDS in constants.php

VALUE
:   ATOM::VALUE() in ATOM\_C.php

VALUE
:   ATOM\_RES::VALUE() in ATOM\_RES.php

VCARD
:   VCARD in VCARD\_C.php

VCARD\_CLASS
:   VCARD::VCARD\_CLASS() in VCARD\_C.php

VCARD\_CLASS
:   VCARD\_RES::VCARD\_CLASS() in VCARD\_RES.php

VCARD\_NS
:   VCARD\_NS in constants.php

VCARD\_RES
:   VCARD\_RES in VCARD\_RES.php

VERSION
:   ATOM::VERSION() in ATOM\_C.php

VERSION
:   ATOM\_RES::VERSION() in ATOM\_RES.php

VERSION\_INFO
:   OWL\_RES::VERSION\_INFO() in OWL\_RES.php

VERSION\_INFO
:   OWL::VERSION\_INFO() in OWL\_C.php

VCARD\_C.php
:   VCARD\_C.php in VCARD\_C.php

VCARD.php
:   VCARD.php in VCARD.php

VCARD\_RES.php
:   VCARD\_RES.php in VCARD\_RES.php

VocabularyClass.php
:   VocabularyClass.php in VocabularyClass.php

VocabularyRes.php
:   VocabularyRes.php in VocabularyRes.php

Vocabulary.php
:   Vocabulary.php in Vocabulary.php

w

top

W3CDTF
:   DC\_RES::W3CDTF() in DC\_RES.php

W3CDTF
:   DC::W3CDTF() in DC\_C.php

WEBLOG
:   FOAF::WEBLOG() in FOAF\_C.php

WEBLOG
:   FOAF\_RES::WEBLOG() in FOAF\_RES.php

WORKPLACE\_HOMEPAGE
:   FOAF\_RES::WORKPLACE\_HOMEPAGE() in FOAF\_RES.php

WORKPLACE\_HOMEPAGE
:   FOAF::WORKPLACE\_HOMEPAGE() in FOAF\_C.php

WORK\_INFO\_HOMEPAGE
:   FOAF\_RES::WORK\_INFO\_HOMEPAGE() in FOAF\_RES.php

WORK\_INFO\_HOMEPAGE
:   FOAF::WORK\_INFO\_HOMEPAGE() in FOAF\_C.php

x

top

XML\_LANG
:   XML\_LANG in constants.php

XML\_NAMESPACE\_DECLARATION\_PREFIX
:   XML\_NAMESPACE\_DECLARATION\_PREFIX in constants.php

XML\_NAMESPACE\_PREFIX
:   XML\_NAMESPACE\_PREFIX in constants.php

XML\_NAMESPACE\_URI
:   XML\_NAMESPACE\_URI in constants.php

y

top

YAHOO\_CHAT\_ID
:   FOAF\_RES::YAHOO\_CHAT\_ID() in FOAF\_RES.php

YAHOO\_CHAT\_ID
:   FOAF::YAHOO\_CHAT\_ID() in FOAF\_C.php

a
b
c
d
e
f
g
h
i
j
k
l
m
n
o
p
r
s
t
u
v
w
x
y
